# Involvement of autophagy in realgar quantum dots (RQDs) inhibition of human endometrial cancer JEC cells

**DOI:** 10.7717/peerj.9754

**Published:** 2020-10-23

**Authors:** Zhengyun Liu, Ke Xu, Yan Xu, Wanling Zhang, Nian Jiang, Shengyu Wang, Guo Luo, Jie Liu, Jinzhu Wu, Huan Wang

**Affiliations:** 1 Key Laboratory of Infectious Disease & Biosafety, Provincial Department of Education, Zunyi Medical University, Zunyi, Guizhou, China; 2 Institute of Life Sciences, Zunyi Medical University, Zunyi, Guizhou, China; 3 Department of Gynecology, Affiliated hospital of Zunyi Medical University, Zunyi, Guizhou, China; 4 Key Laboratory for Basic Pharmacology of Ministry of Education, Zunyi Medical University, Zunyi, China; 5 Department of Chemistry, School of Science, Harbin Institute of Technology, Harbin, China

**Keywords:** Arsenic and RQDS, Endometrial cancer cells, Autophagy, Transmission electron microscopy, EGFP-LC3 plasmid transfection

## Abstract

Realgar (As_4_S_4_) has been used in traditional Chinese medicines for treatment of malignancies. The poor solubility of As_4_S_4_ hampered its clinical applications. Realgar quantum dots (RQDs) were developed to overcome these problems. Previous studies revealed that the RQDs were effective against endometrial cancer JEC cells and hepatocarcinoma HepG2 cells via inducing apoptosis.Apoptosis and autophagy are important programmed cell death pathways leading to anticancer effects. This study further examined effects of RQDs on autophagy, focusing on the formation of the autophagosome in JEC cells. CCK8 assay was used to examine cell proliferation. Flow cytometry was used to analyze cell cycle. Transmission electron microscopy (TEM) was used to examine the autophagy, cells were transfected with pEGFP-C3-MAP1LC3B plasmid to examine effects of RQDs on autophagosome via confocal microscope. Autophagy-related proteins were examined by Western blot. RQDs exhibited cytotoxicity in JEC cells in a concentration- and time- dependent manner. RQDs induced G2 and S phase arrest in JEC cells. RQDs significantly induced autophagy, with the double-membrane and autophagosome-like structures by TEM. The diffused distribution of pEGFP-C3-MAP1LC3B green fluorescence were become the punctuate pattern fluorescence after treatment with RQDs in cells transfected with pEGFP-C3-MAP1LC3B plasmid RQDs increased the expression of autophagyregulatory proteins LC3 I/II, Beclin-1, p62 and Atg12 in a concentration-dependent manner, similar to autophagy induced by serum starvation, except for p62, as induction of p62 is a characteristic of arsenic compounds. Taken together, the present study clearly demonstrated that RQDs can induce autophagy in JEC cells as one of mechanisms of anticancer effects, and indicated that RQDs may be developed as an autophagy inducer.

## Introduction

Mineral arsenicals have along history of therapeutic use in traditional medicines (Wang et al., 2017; Wu et al., 2011), and are still used today based on Pharmacopeia of China (Pharmacopeia, 2015). Arsenic trioxide (As_2_O_3_) is a good example of how arsenic is identified, purified, and successfully used to treat with cancers. FDA approved As_2_O_3_ for clinical application in the treatment of acute promyelocytic leukemia (APL) in 2000 (Hu et al., 2014). Realgar contains > 90% arsenic sulfide (As_4_S_4_), and has been widely used in traditional Chinese medicines for more than 2000 years in the treatment of malignant diseases, as well as syphilis, parasitic infections, malaria,psoriasis and cancer (Liu et al., 2008). Realgar (As_4_S_4_) nanoparticles, arsenic trioxide (As_2_O_3_) and arsenic sulfide induced autophagy and apoptosis in human melanoma cells osteosarcoma cells. So, induced autophagy and apoptosis were major mechanisms in realgar anti-tumor effects (Pastorek et al., 2014; Wang et al., 2017).

The poor solubility of realgar hampered its clinical applications (Ding et al., 2015; Wu et al., 2011). It is clear that solubility and bioavailability of realgar are poor as compared to As_2_O_3_, thus limiting its efficacy against cancer cells (Liu et al., 2008). Several means have been used to improve the solubility of realgar. The realgar bioleaching solution (Xie et al., 2014), realgar nanoparticles (Tian et al., 2014), and realgar quantum dots (RQDs) (Wang et al., 2015a) were developed to overcome these problems.

The water-soluble-As_4_S_4_ showed much higher cytotoxicity towards HL-60 leukemia cells in vivo and in vitro studies than raw-As_4_S_4_ (Ma et al., 2016). Milling realgar into nanoparticles has more potent anti-multiple myeloma (MM) activities than arsenic trioxide (ATO), with enhanced depletion of MM stem-like SP cells and synergistic anti-MM activity with lenalidomide and melphalan (Cholujova et al., 2017). Compared with the crude realgar powders, smaller size of realgar has higher solubility, increased bioavailability and anti-tumor effect (An et al., 2011; Wu et al., 2011).

RQDs (5.48 ± 1.09 nm) were prepared by a wet chemical method to make it soluble in water (Wang et al., 2015a; Wang et al., 2015b). Our previous studies revealed that the RQDs were effective against human endometrial cancer JEC cells and hepatocellular carcinoma HepG2 cells, and reduced carcinoma size of uterine cervix in tumor-bearing mice (Qin et al., 2015; Wang et al., 2015a; Wang et al., 2015b). In brief, RQDs induced apoptosis and necrosis in JEC cells, induced ER stress and the loss of mitochondrial membrane potential in HepG2 cells.

Macroautophagy (hereafter called autophagy) is an evolutionarily conserved lysosomal degradation process crucial for cellular homeostasis and adaptation to stress. It is tightly controlled by highly conserved molecules called autophagy-related proteins; excessive autophagy in fact may destroy essential intracellular molecules and structures to a level incompatible with cell life (Murrow & Debnath, 2013). In human cancer, autophagy has become a potential target for anti-cancer drugs because it can be a mechanism of non-apoptotic cell death (type II programmed cell death) (Choi, Ryter & Levine, 2013).

Apoptosis and autophagy are important programmed cell death processes that maintain organism and cellular homeostasis. Apoptosis can be initiated via extrinsic and intrinsic pathways dismantling damaged or unwanted cells; autophagy maintains cellular homeostasis through recycling selective intracellular organelles and molecules. Yet in some conditions, autophagy can lead to cell death, indicates that some crosstalk between apoptosis and autophagy (Fan & Zong, 2013; Yuan et al., 2018).

The goal of the present study is to determine the role of autophagy in RQDs anti-tumor effects, using flow cytometry analysis, TEM, confocal microscopy and Western blot, and the results, especially from TEM and confocal microscopy, clearly demonstrated the involvement of autophagy in RQDs antitumor effects.

## Materials and Methods

### Cell culture

Human endometrial cancer JEC cells were provided by Zhongmin Wu (Zunyi Medical University). The cells were maintained in DMEM medium supplemented with 10% FBS, 100 IU/mL penicillin and 100 g/mL streptomycin at 37C 5% CO_2_.

### Reagents and plasmids

RQDs were prepared by the previous method by Dr Jin-Zhu Wu from Harbin Institute of Technology (Harbin, China) (Qin et al., 2015; Wang et al., 2015a; Wang et al., 2015b). The realgar powder (2.0 g) was dispersed into 50 mL ethanolamine under bubbling argon gas and dissolving with ultrasonic method, 1,500 rpm for 5 min to collect supernatant. Following centrifugation, the supernatant will be heated at 80 °C for 12 h, added saturated water solution of citric acid in supernatant until pH 8.0 ± 0.2 and kept for another 36 h at same temperature. Finally, determination the photoluminescence (PL) quantum yield by a previous method.

Cell Counting Kit-8 (CCK-8) was purchased from Yiyuan Biotechnologies (Guangzhou, China). DMEM was purchased from Thermo Fisher Scientific (Massachusetts, USA). FBS was purchased from Gibco Life Technologies (New York, USA). Cell Cycle Detection Kit was from KeyGEN Bio-TECH (Jiangsu, China). Antibodies LC3I/II, Beclin-1, P62, Atg5, Atg7, Atg16L1, Atg12 and GAPDH were from Cell Signaling Technology (Boston, USA). Secondary antibodies Goat anti-rabbit IgG (H + L) and Goat anti-mouse IgG (H + L) were purchased from Thermo Fisher Scientific.The pEGFP-C3-MAP1LC3B plasmid was purchased from Biogot Biotechnology (Nanjing, China).

### CCK8 assay to determination cell proliferation

The cell viability after treated with RQDs was measured using CCK-8 assay. Cells (1 ×10^4^) were plated in 96-well plates and incubated 24 h in cell culture medium to allow them to adhere. Cells were then exposed to different concentrations of drug or vehicle for up to 72 h. Cell viability was evaluated by the CCK-8 assay. The absorbance at 450 nm was measured with a microplate reader. Cell viability was expressed as a percentage of vehicle control.

### Flow cytometric to analysis of cell cycle

The JEC cells were plated at a concentration of 5 ×10^5^ cells/well in 6-well plates for 24 h and incubated with RQDs for 24 h. The cells were collected, washed with PBS, and then re-suspended in 70% methanol for overnight at 4 °C. The cells were washed twice with PBS and re-suspended in RNase A buffer (100 μ L) for 30 min at 37 °C. Then, 400 μ L propidium iodide (PI) was add to the mixture for 10 min in the dark. The cell cycle was detected using flow cytometric after staining.

### Transmission Electron Microscopy to observed autophagosome

### -like structures

The JEC cells were plated at a concentration of 5 ×10^6^ cells/well in 15-cm dish for 24 h and incubated with RQDs for 24 h. The cells were collected and fixed in 3% glutaraldehyde, 0.1 mol/L cacodylate buffer (pH7.4) for 1 h at 4 °C, after washing with PBS, the cells were post-fixed in OsO4 and embedded in epoxy resin. The 50-nm thin sections were stained with uranyl acetate/lead citrate (Lu et al., 2014). The cells were observed and photographed under the TEM.

### Confocal microscopy examination of the expression of EGFP-LC3

JEC cells (4 ×10^5^) were seeded in 35-mm dishes for 24 h, and the plasmid pEGFP-C3-MAP1LC3B were transiently transfected into cells with Lipofectamine 2000 transfection reagent according to the manufacturer’s instructions. After 48 h, the cells were treated with RQDs for 24 hand examined under confocal microscope.

### Western blot to determination autophagy regulatory proteins

Western blot sample were collected in RIPA lysis buffer, BCA assay was used to determined cell protein concentrations. The protein was resolved on a SDS-PAGE, transferred to PVDF membrane and blocked with 5% BSA for 2 h at room temperature. The blot were then probed with primary antibodies (LC3I/II, Beclin-1, P62, Atg5, Atg7, Atg16L1, Atg12 and GAPDH) overnight at 4 °C, washed six times with 1 × TBST in 30 min. Followed by fluorescence secondary antibodies at room temperature for 2 h, and the protein were visualized and quantified by using Odyssey infrared imaging system (Nebraska, USA).

### Statistical analysis

All of the data were expressed as the mean ± SE, and analyzed with the SPSS 17.0 software, one-way ANOVE followed by LSD multiple range test or Dunnett’s T3 (3). The *P*-value < 0.05 was statistically significant.

## Results

### RQDs inhibited the cells growth in JEC cells

**Figure 1:**
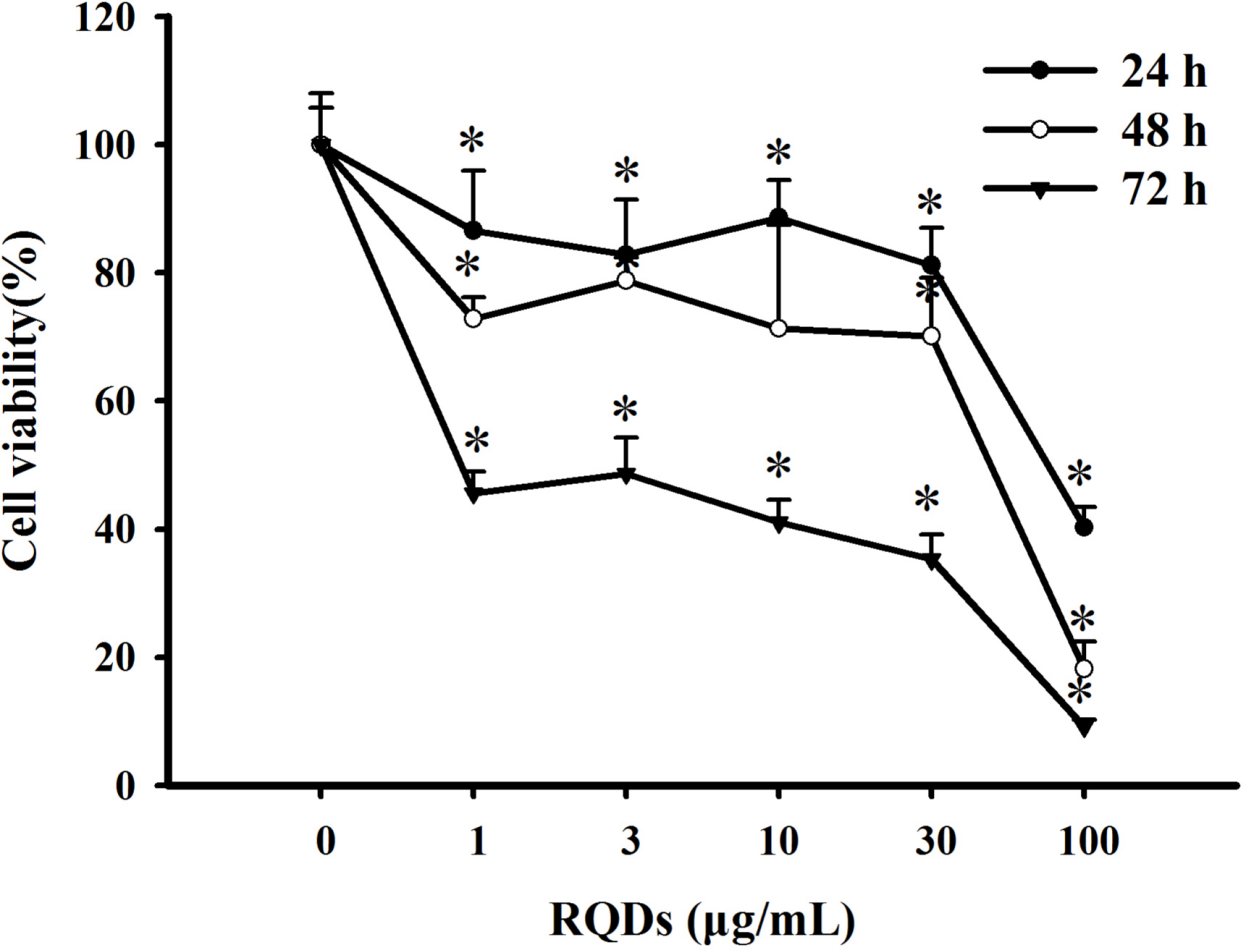
Growth inhibition effects of RQDs on JEC cells. Cells were treated with various concentrations of RQDs (0, 1, 3, 10, 30 and 100 µg/mL) for 24 h, 48 h, 72 h and assessed for viability using the CCK8 assay. The data were expressed as the mean ±  SE of three experiments. ^∗^*P* < 0.05 indicated a significant difference from the control group.

The CCK8 assay was used to investigated the effect of RQDs on viability of JEC cells. As shown in Fig. 1,cell viability significantly decreased as the RQDs dose(0, 1, 3, 10, 30 and 100 μ g/mL) or exposure time (24 h, 48 h and 72 h) increased. The IC_50_ were 48.92 μ g/mL (24 h), 44.88 μ g/mL (48 h), 2.85 μ g/mL (72 h), consistent with previous research (Wang et al., 2015a; Wang et al., 2015b).

### RQDs arrested cell cycle in G2 and S phase

**Figure 2:**
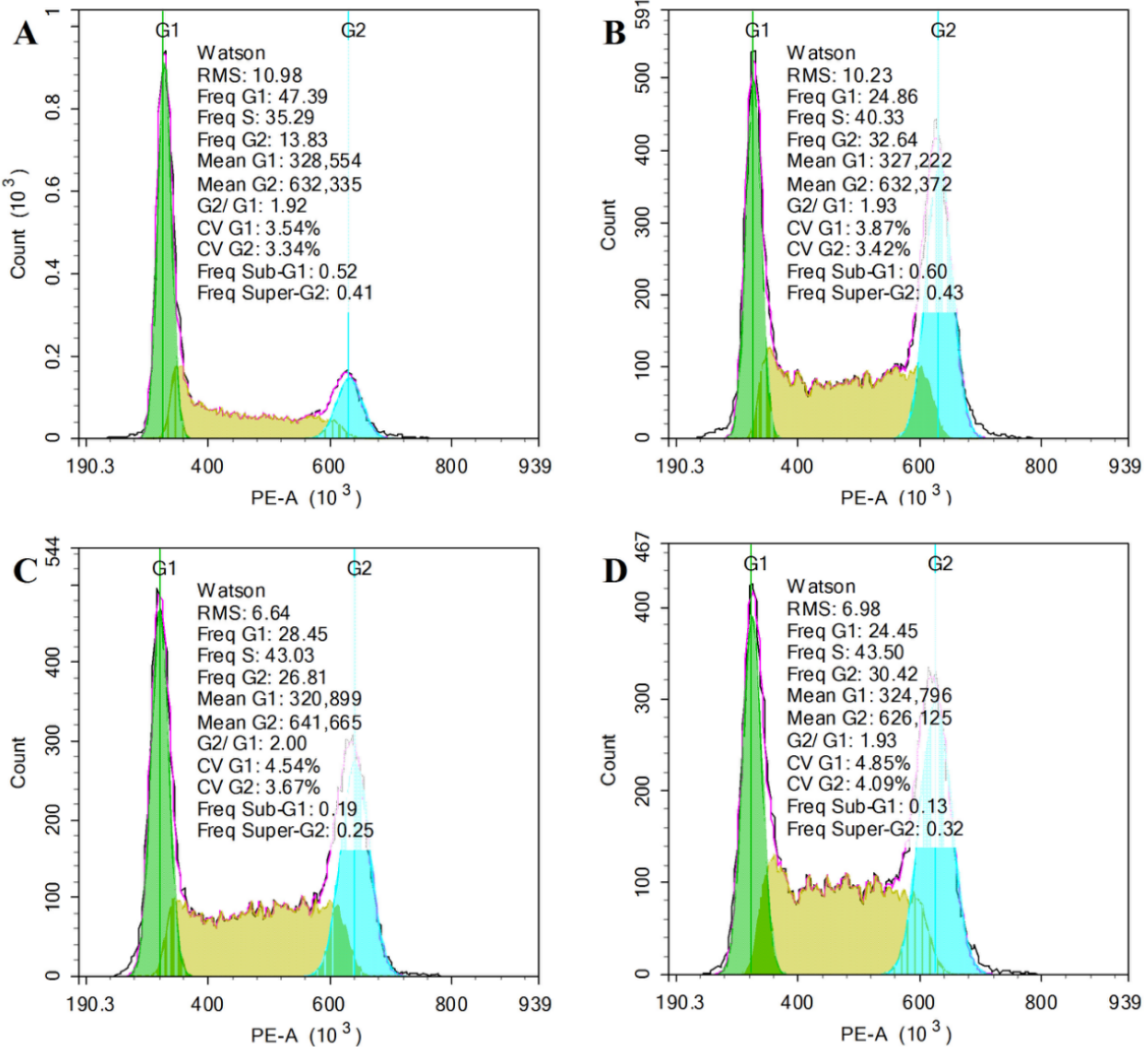
RQDs arrest cell cycle in G2 and S phase. Cells were treated for 24 h with RQDs at 0 (A), 10 (B), 20 (C) and 40 (D) µg/mL and cell cycle phase distribution was determined by flow cytometry.

The Cell Cycle Detection Kit was used to detected the effect of RQDs on JEC cells by flow cytometry. The IC_50_ of RQDs was 48.92 μ g/mL (24 h), so we chose the concentration (0, 10, 20 and 40 μ g/mL) under the IC_50_ to carry out the experiment. Following 24 h of treatment with 40 μ g/mL RQDs, population of cells in G1 phase was significantly reduced from 49.96% to 25.69%, cells in the G2 phase increased appreciably from 14.41% to 29.43%, and cells in S phase increased from 33.26% to 43.50% (Table 1). Thus, RQDs influenced the cell cycle to arrest cells in the G2 and S phase, especially in G2 phase (Fig. 2).

**Table 1:** RQDs influenced the cell cycle in JEC cells.

RQD (µg/mL)	G1(%)	S(%)	G2(%)
0	49.96 ± 2.16	33.26 ± 1.76	14.41 ± 1.04
10	28.55 ± 3.25^*^	38.37 ± 1.81	29.78 ± 2.51^*^
20	27.48 ± 0.85^*^	38.14 ± 4.23	32.44 ± 4.89^*^
40	25.69 ± 2.49^*^	43.50 ± 3.18^*^	29.43 ± 5.12^*^

**Notes.**

The data were expressed as the mean SE of three experiments.

* *P* < 0.05 indicated a significant 3 difference from the control group.

### RQDs induced autophagy in JEC cells

**Figure 3:**
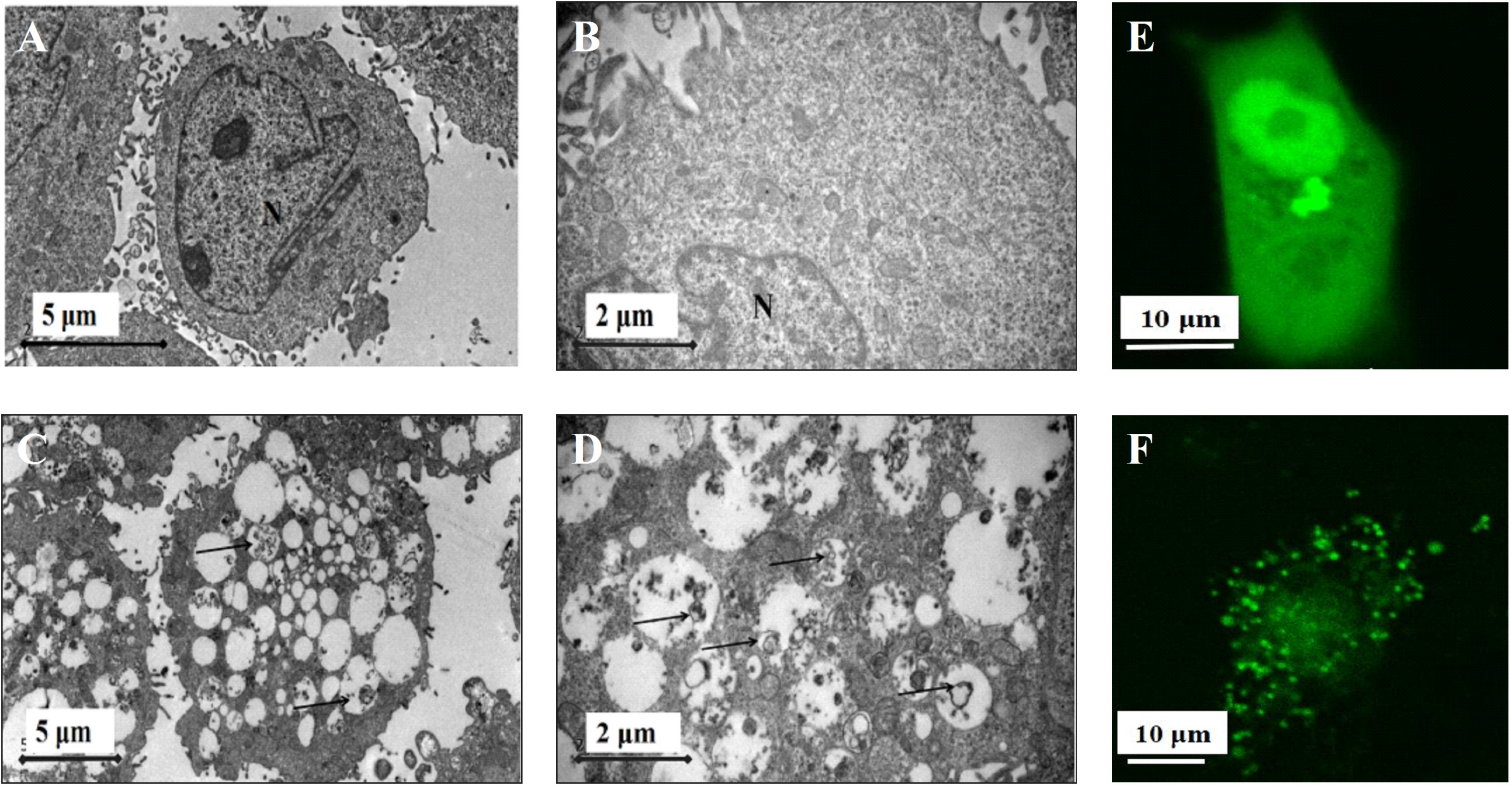
RQDs induced autophagy in JEC cells. Cells were treated for 24 h with RQDs 40 µg/mL (C, D) and ultrastructure was determined by TEM; A, B were treated with RQDs 0 µg/mL (N, nucleus. Arrowhead, autophagosomes) (A, C). Cells were seeded in 35-mm perti dishes, transiently transfected pEGFP-C3-MAP1 LC3B for 48 h, treated with 40 µg/mL RQDs for 24 h (F); E was treated with RQDs 0 µg/mL, analyzed with confocal microscopy images (E, F).

The hallmark of autophagy induction is the formation of cellular autophagosome punctuate containing the microtubule-associated protein LC3I/II. Translocation of LC3 protein from the cytosol (LC3-I form) to the autophagosome membrane (LC3-II form) is currently a standard method to monitor autophagy (Kulkarni et al., 2016). This phenomenon was confirmed by transmission electron microscopy (TEM) analysis of JEC cells with 40 μ g/mL RQDs for 24 h showing the double-membrane and autophagosome-like structures (Figs. 3C, 3D).

A green fluorescent pEGFP-C3-MAP1LC3B plasmid was transiently transfected into JEC cells and examined with confocal microscopy. This protein normally exhibits diffused cytosolic distribution in autophagy (Tanida, Ueno & Kominami, 2004). As shown in Fig. 3, diffused distribution of pEGFP-C3-MAP1LC3B in the basal state was observed in the transfected cells (Fig. 3E). After treatment with RQDs, punctuate patterns of pEGFP-C3-MAP1LC3B represented autophagic vacuoles in the whole cytoplasm (Fig. 3F).

### RQDs altered the expression of LC3 and autophagy regulatory proteins

**Figure 4:**
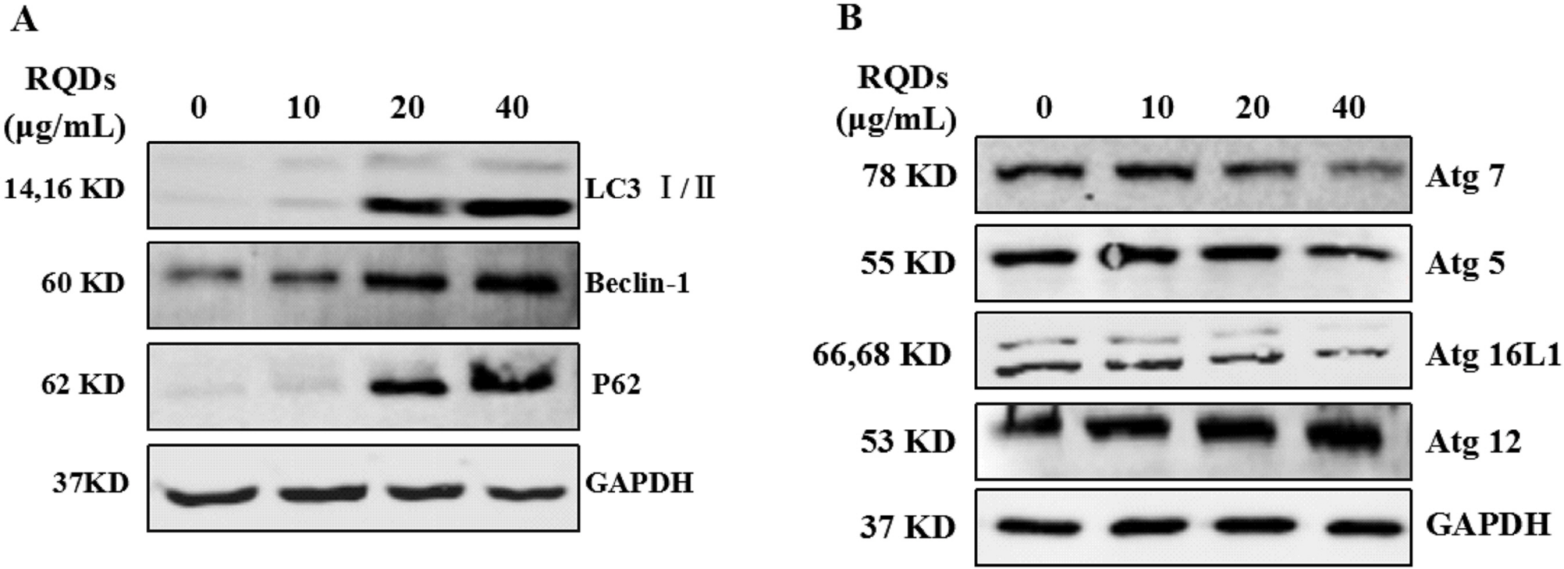
RQDs alter the expression of (A) LC3I/II and (B) modulate autophagy regulatory proteins. Cells were treated with increasing concentration of RQDs (0, 10, 20 and 40 µg/mL) for 24 h. GAPDH was used as the loading control.

Next, we test the expression of LC3 I/II after treat with RQDs and found that LC3 I/II protein dramatically increase, especial the LC3 ? (Fig. 4A). Beclin-1 is a part of the class ? phosphatidylinositol 3-kinase (PtdIns3K) complex and is required for the initiation of the autophagosome formation in autophagy. Human PIK3C3/VPS34 is the catalytic subunit of PtdIns3K (Zhang et al., 2015). p62 is a multi-domain protein that interacts with cargos for autophagicde gradation as well as several key signaling components (Moscat & Diaz-Meco, 2012). In our study, Beclin-1 and p62 were up-regulated after RQDs treated (Fig. 4A). Autophagy is characterized by the formation of double membrane vesicles called autophagosomes that after engulfing cellular components fuse with lysosomes resulting in degradation of cargo. Critical components of the autophagy pathway include Atg5, Atg7, Atg16L1 and Atg12, which are involved in the elongation and closure of the autophagosomes membrane. RQDs also modulate autophagy regulatory proteins. We observed the expression of Atg7, Atg5 and Atg16L1 were decreased at the highest concentration of RQDs, but RQDs induced the expression of Atg12 (Fig. 4B).

Serum starvation for 48 h also induced the expression of LC3 I/II and Beclin-1, but the expression of p62 was decreased (Fig. S1). These results indicate that the cells underwent autophagy after RQDs but were different from starvation (p62 increase).

We have used autophagy inhibitors 3-MA and autophagy inducer rapamycin to study RQDs’ effect and found the effect of RQDs induced autophagy cannot be repressed by 3-MA, nor enhanced by rapamycin (Fig. S2), Suggesting that RQDs may be a special autophagy inducer (Zhao et al., 2019).

## Discussion

The major novel findings in the present study are the clear evidence of RQD-induced autophagy via morphological approaches. TEM assay showed RQDs (40 μ g/mL) could significantly induce autophagy, and the diffused green fluorescence were become the dot green fluorescence after treatment with RQDs (40 μ g/mL) in JEC cells transiently transfected with pEGFP-C3-MAP1LC3B plasmid. Furthermore, LC3?/?, Beclin-1and p62 proteins were increased with RQDs (0–40 μ g/mL) treatment. These results clearly demonstrated the involvement of autophagy in RQD antitumor effects.

Realgar and realgar nanoparticles have been demonstrated to inhibit the proliferation of several cancer cell lines, including cancer stem-like cells (CSLCs) in lung cancer (Chang et al., 2016) and HPV16-positive cervical cancer cell line SiHa and HPV16-positive immortalized cervical epithelial cell line S12 (Ding et al., 2018). RQDs had been demonstrated to inhibit cell growth in JEC cells and HepG2 cells with CCK8 assay (Qin et al., 2015; Wang et al., 2015a; Wang et al., 2015b). In Adriamycin-selected and P-gp positive multidrug-resistant human leukemia cell K562/ADM and its parental K562 cells (Wang et al., 2018), human melanoma celllines (BOWES and A375) (Pastorek et al., 2014) and human osteosarcoma cell lines (143B, MG-63, HOS and U2OS) (Wang et al., 2017), realgar induced cell cycle arrest or induced cell autophagy and apoptosis. In our previous studies, RQDs induced apoptosis in JEC cells, and cell cycle arrest was closely related to apoptosis. According to flow cytometric analysis, we found RQDs could cause G2 and S cell phase arrest in JEC cells.

Some studies have focused on a putative correlation between cell-cycle progression and autophagy ?ux, clearly suggesting a strong correlation between autophagy activation and the induction and possibly execution of cell-cycle arrest programs, as well as autophagy regulation of the cell division process (Mathiassen, Zio & Cecconi, 2017), We have found RQDs arrested cell cycle in G2 and S phase, predicting RQDs had influence on the autophagy. Due to the small size of sub-cellular structures participating in the autophagy process, TEM remains a prominent position in the methodological arsenal for studying autophagy (Kovacs, 2015) after the first discovery of autophagy by TEM 50 years ago (Eskelinen et al., 2014). The higher magnification image could clearlyshow the presence of autophagic vacuoles containing partiallydegraded cytoplasmic material and exhibiting increased electrondensity by TEM (Lin et al., 2014). In the present study, we found that RQD induced double-membrane and autophagosome-like structures with TEM. The pEGFP-C3-MAP1LC3B plasmid was normallyexhibits diffused cytosolic distribution in autophagy, we found the punctuate patternsof pEGFP-C3-MAP1LC3B after treatment with RQDs in confocal microscopy, consistent with TEM observations.

In autophagy, 2 ubiquitin-like (UBL) conjugation pathways Atg12 and Atg8 (LC3 in mammals), are required for early stage of autophagosome biogenesis. One of which the Atg12 conjugated to Atg5,which is activated by the E1-like enzyme Atg7 and transferred to the E2-like enzyme ATG10 (Mizushima et al., 1998). The Atg12 complex (Atg12-Atg5) is conjugated to the Atg16 (Atg16L1 in mammals). TECPR1 forms a complex with the Atg12–Atg5 conjugate and exclusively with Atg16L1 in mammalian autophagosome maturation whereas it co-localizes with the Atg12–Atg5-Atg16L1 complex to targeting bacterial pathogens in selective autophagy (Chen et al., 2012; Chen & Zhong, 2012). In our study, LC3 I/II, Beclin-1, p62 and Atg12 were up-regulated after RQDs treated, and the expression of Atg7, Atg5 and Atg16L1 were decreased at the highest concentration of RQDs. Accumulation of P62 means that autophagy was been inhibited and degradation was reduced, eventually leading to autophagy accumulation. So, the RQDs inhibited the degradation of autophagy (Mathew et al., 2009).

Autophagic vacuoles might be normal constituents of healthy cells sequestering and degrading cytoplasm in response to the metabolic demands during starvation (Cd, 1964). Autophagy can serve as a survival mechanism during nutrient deprivation or metabolic stress, whereas it can also lead to cellular death (Baehrecke, 2005), serum starvation can induces cell autophagy. After serum starvation, the levels of Beclin-1 and LC3-II in serum-starved cells are increased compared with serum culture, but  expression  of 62 is  reduced (Wang et al., 2015a; Wang et al., 2015b; Yang et al., 2017). Similar, we found in serum-starved JEC cells for 48 h LC3I/II and Beclin-1 were increased, and the p62 was decreased. Similar to serum-starvation, autophagy inducer rapamycin also down-regulated the expression of P62 (Li et al., 2017). However, autophagy induction by arsenic compounds is an exception (Bolt et al., 2012; Lau et al., 2013), arsenic induces p62 up-regulation in vitro and in vivo and induces LC3 I/II up-regulation in vitro (Shah et al., 2017). In our study, we found the expression of P62was up-regulated after RQDs treatment, which was different from the classical autophagy inducer (rapamycin) and the classical autophagy induction method (serum starvation), both of which decreased the expression of P62, while RQDs induced the expression of P62, which is unique for arsenic compounds.

In our previous studies, RQDs induced apoptosis and necrosis in JEC cells, induced ER stress and the loss of mitochondrial membrane potential in HepG2 cells. Now, we found RQDs induced autophagy in JEC cells. Apoptosis and autophagy were important in development and normal physiology and in a wide range of diseases. The antitumor mechanisms of RQDs include the induction of both autophagy and apoptosis, because when autophagy was not under control, it will be followed by apoptosis.

## Conclusion

In summary, the present study clearly demonstrated that the autophgy can be induced by RQDs in JEC cells, as evidenced by TEM, confocal image of pEGFP-C3-MAP1LC3B, and autophagy-related proteins. Induction of autophagy could be one of RQDs anticancer mechanisms, indicate that RQDs may be developed as a newly autophagy inducer.

##  Supplemental Information

10.7717/peerj.9754/supp-1 Supplemental Information 1 Serum-starved induced the expression of autophagy regulatory proteins Cells were treated in serum-starved for 48 h. GAPDH was used as the loading control.

10.7717/peerj.9754/supp-2 Supplemental Information 2 The effect of 3-MA and rapamycin in RQDs induced autophagy Cells were treated with increasing concentration of 3-MA (2.5 and 5 nM) or rapamycin (Rap) (10 and 20 mM )with RQDs 40 µg/mL for 24 h. GAPDH was used as the loading control.

10.7717/peerj.9754/supp-3 Supplemental Information 3 Raw data for Fig. 1

10.7717/peerj.9754/supp-4 Supplemental Information 4 Raw data for Fig. 2

10.7717/peerj.9754/supp-5 Supplemental Information 5 Raw data for Fig. 3

10.7717/peerj.9754/supp-6 Supplemental Information 6 Raw data for Fig. 4

10.7717/peerj.9754/supp-7 Supplemental Information 7 Raw data for Fig. S1

10.7717/peerj.9754/supp-8 Supplemental Information 8 Raw data for western blot

## Additional Information and Declarations

### Data Availability

The following information was supplied regarding data availability: The raw measurements are available in the Supplementary Files.
