# Supplementary figures and images for: Involvement of autophagy in realgar quantum dots (RQDs) inhibition of human endometrial cancer JEC cells
Source: PeerJ. 2020 Oct 23;8:e9754. doi: 10.7717/peerj.9754 (PMC7587054; doi:10.7717/peerj.9754)

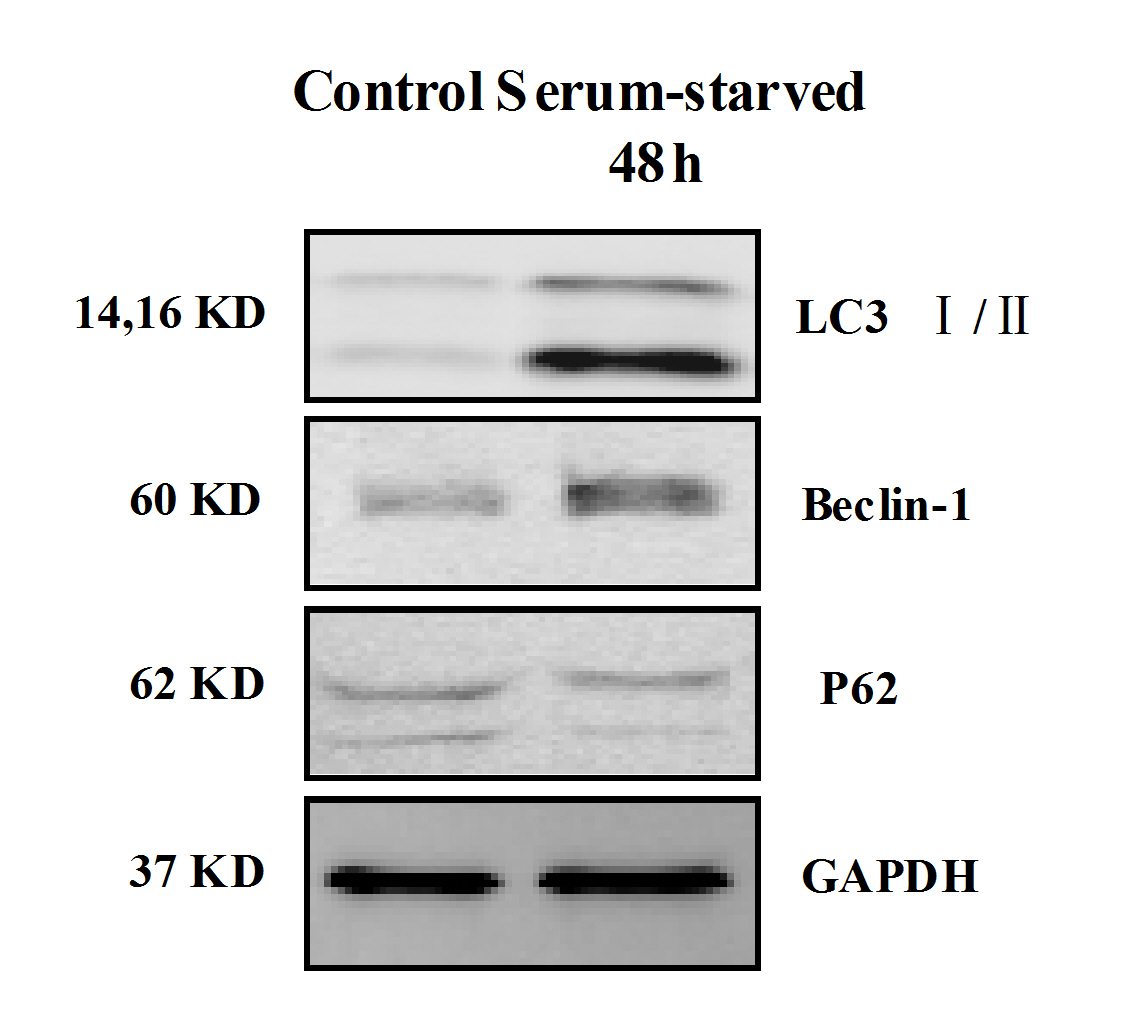

Supplement: Supplemental Information 1 — Cells were treated in serum-starved for 48 h. GAPDH was used as the loading control. [file peerj-08-9754-s001.jpg]

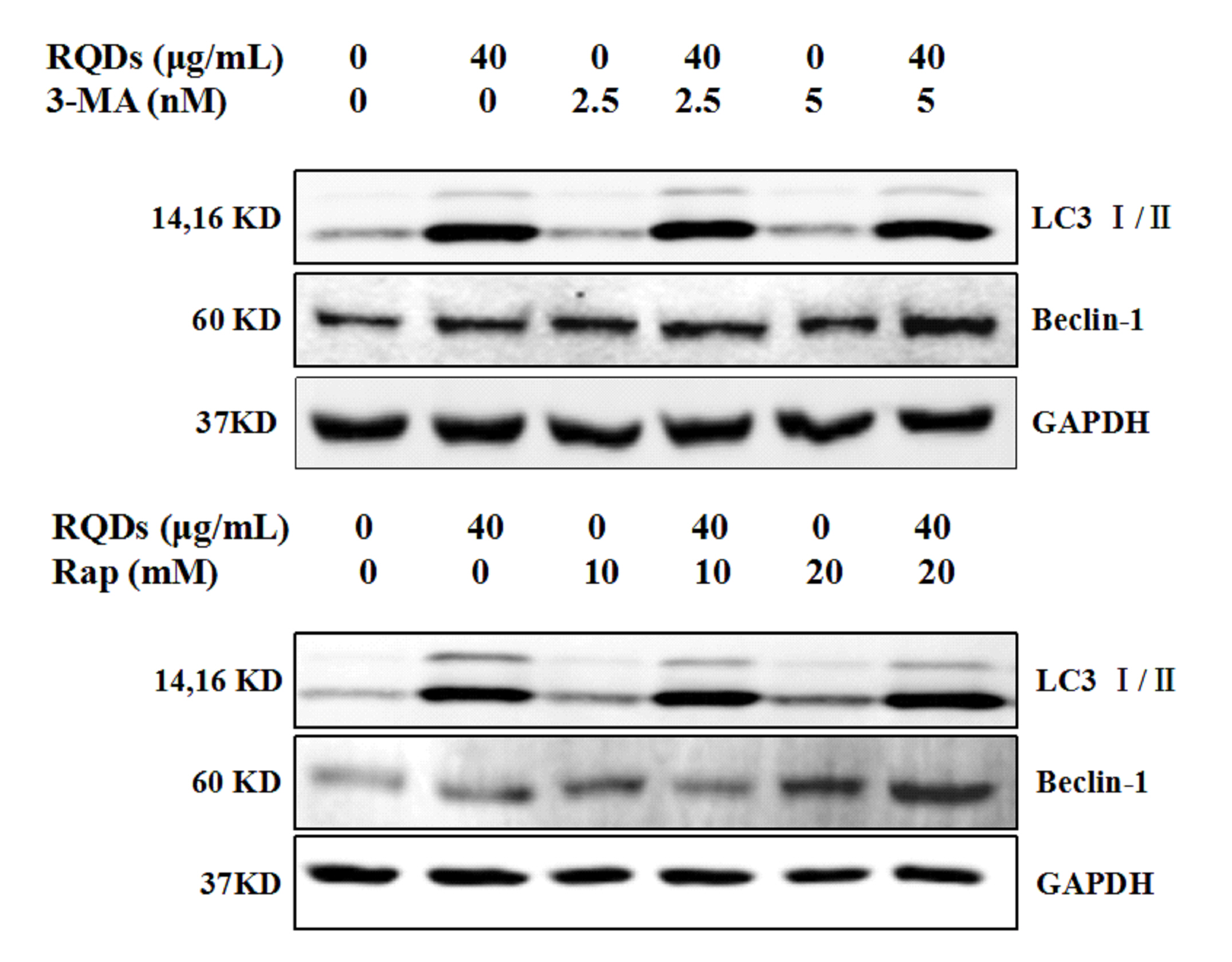

Supplement: Supplemental Information 2 — Cells were treated with increasing concentration of 3-MA (2.5 and 5 nM) or rapamycin (Rap) (10 and 20 mM )with RQDs 40 µg/mL for 24 h. GAPDH was used as the loading control. [file peerj-08-9754-s002.jpg]

## Slide 1
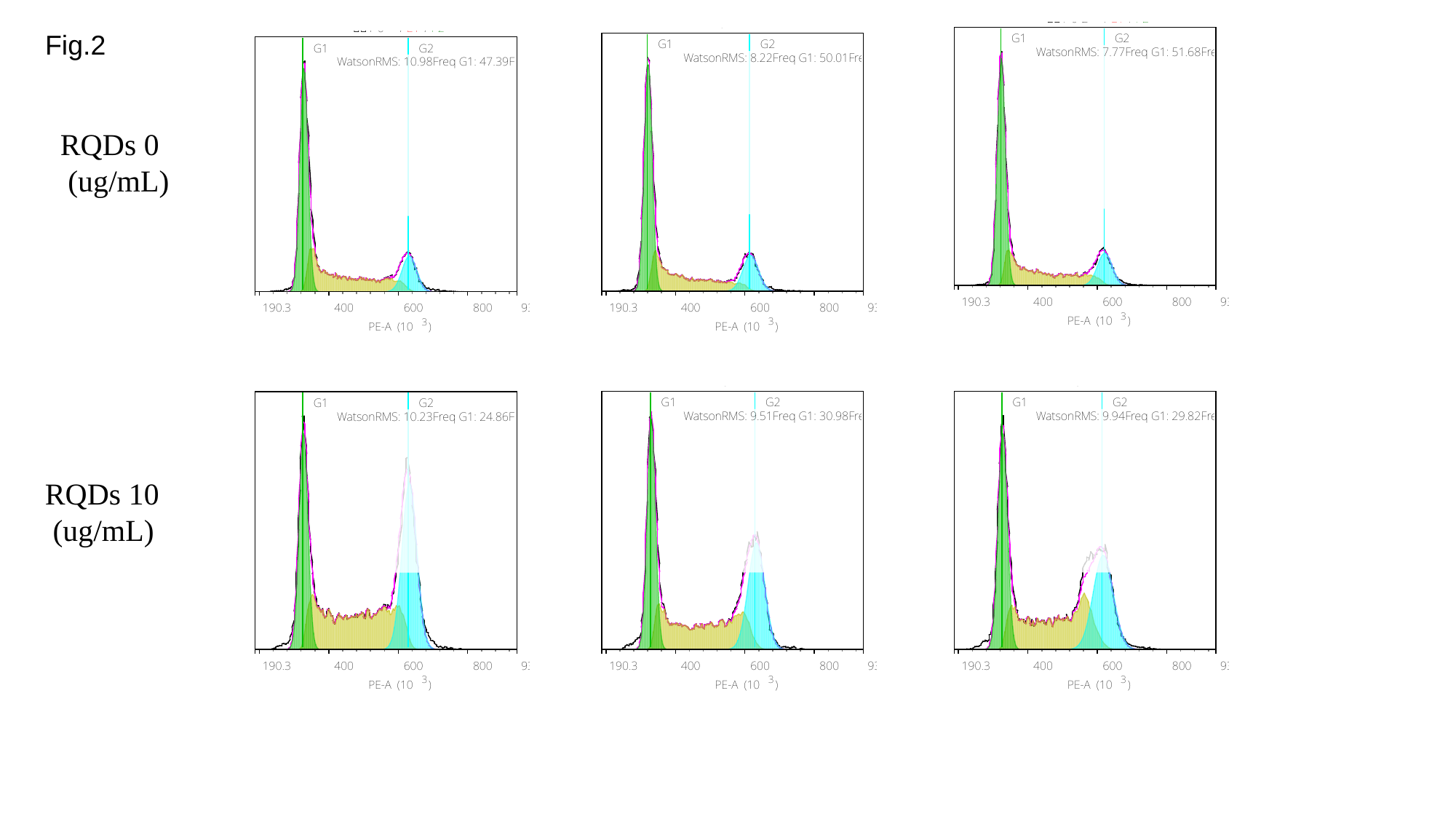

Fig.2
RQDs 0
 (ug/mL)
RQDs 10
 (ug/mL)

## Slide 2
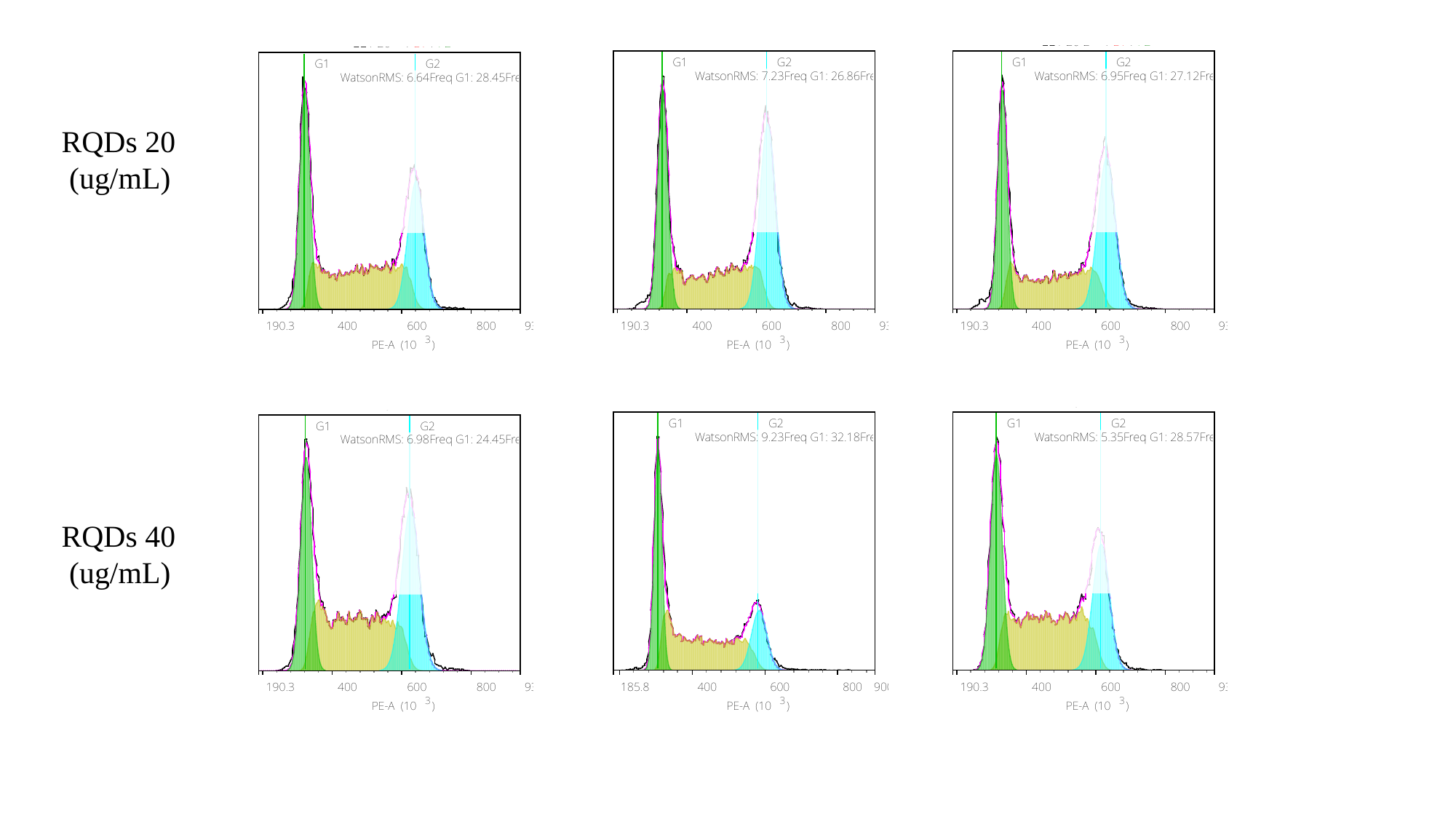

RQDs 20
 (ug/mL)
RQDs 40
 (ug/mL)

Supplement: Supplemental Information 4 [file peerj-08-9754-s004.pptx]

## Slide 1
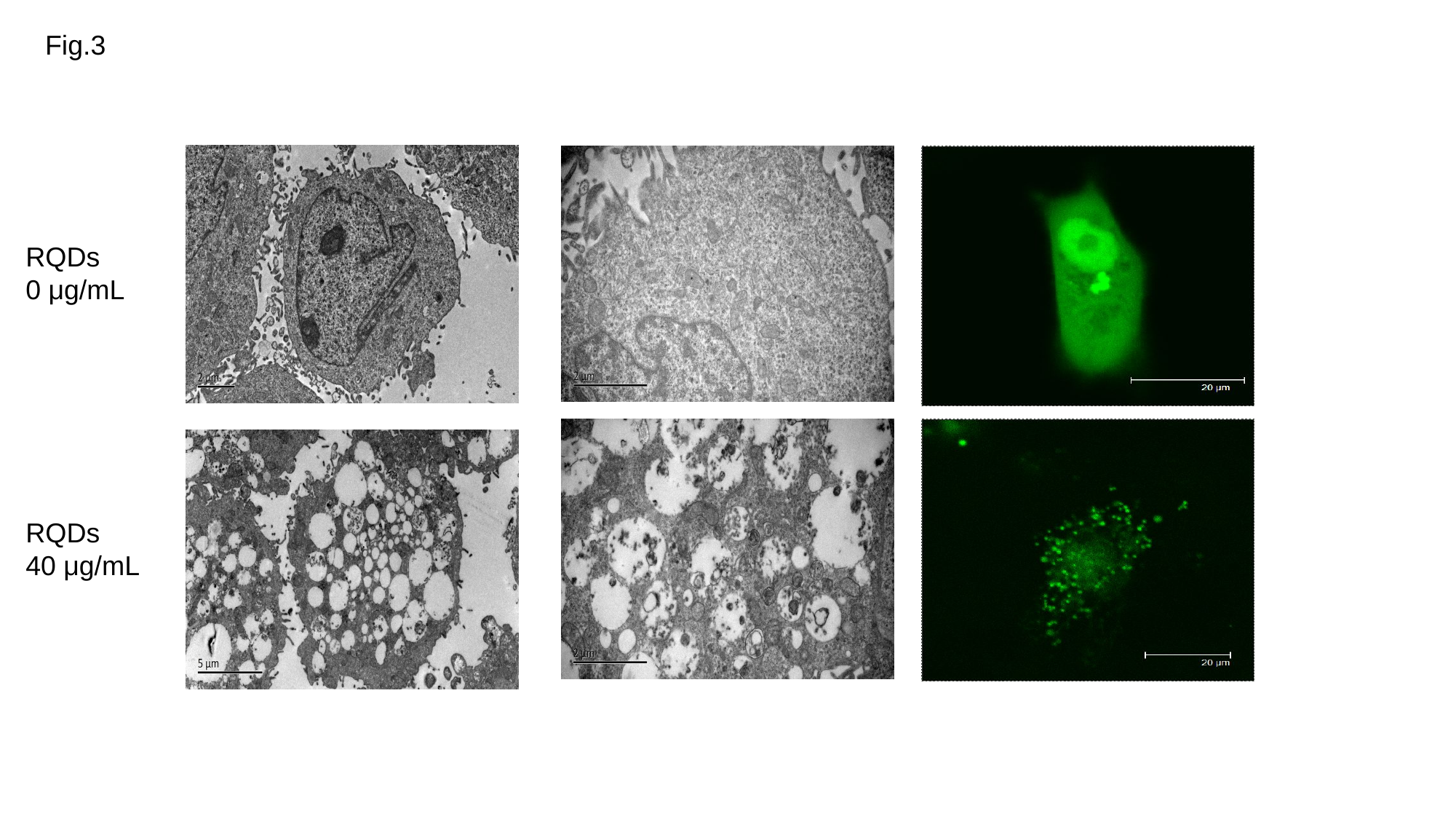

Fig.3
RQDs
0 μg/mL
RQDs
40 μg/mL

Supplement: Supplemental Information 5 [file peerj-08-9754-s005.pptx]

## Slide 1
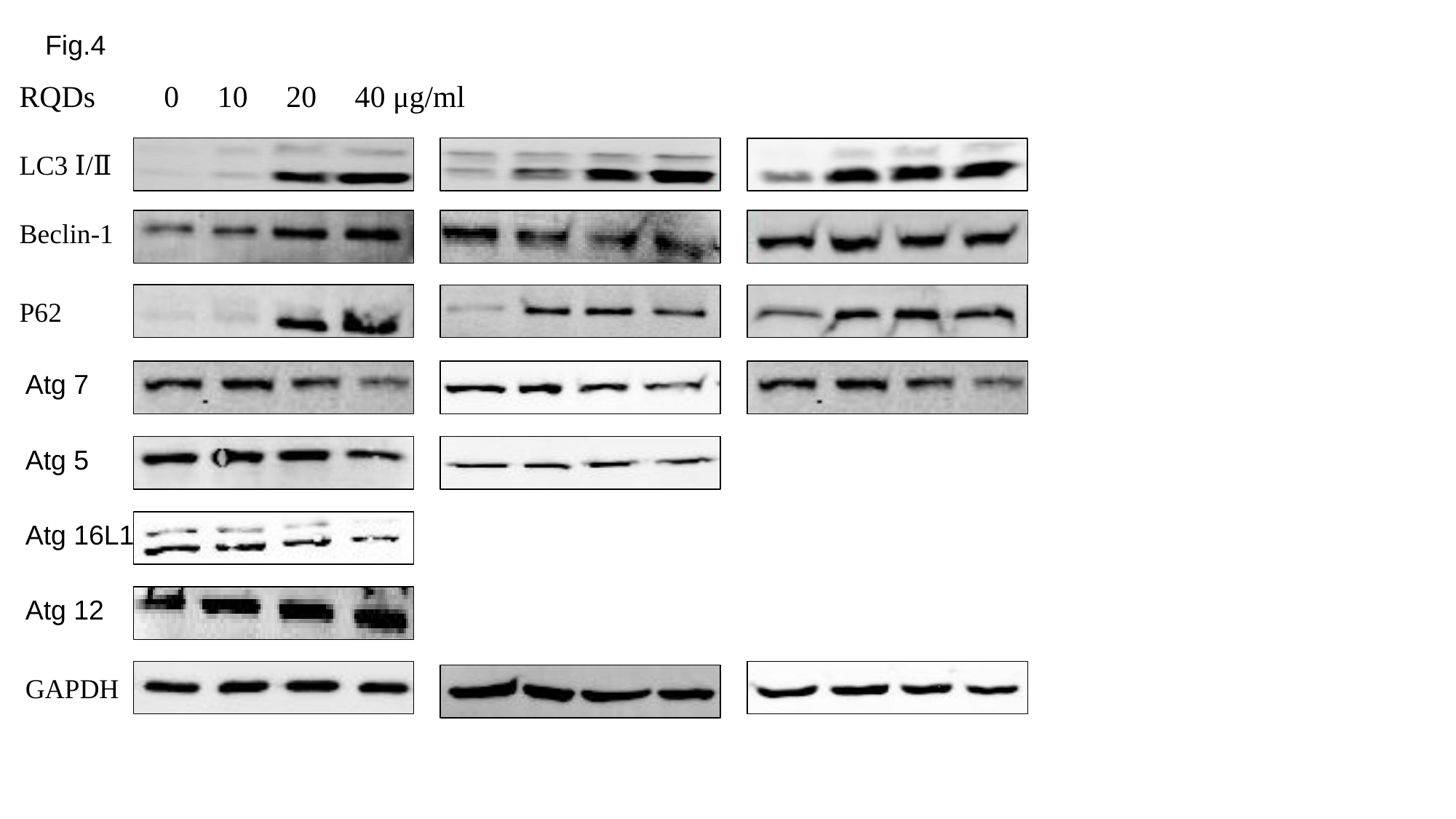

Fig.4
RQDs 0 10 20 40 μg/ml
LC3 Ⅰ/Ⅱ
Beclin-1
P62
Atg 7
Atg 5
Atg 16L1
Atg 12
GAPDH

Supplement: Supplemental Information 6 [file peerj-08-9754-s006.pptx]

## Slide 1
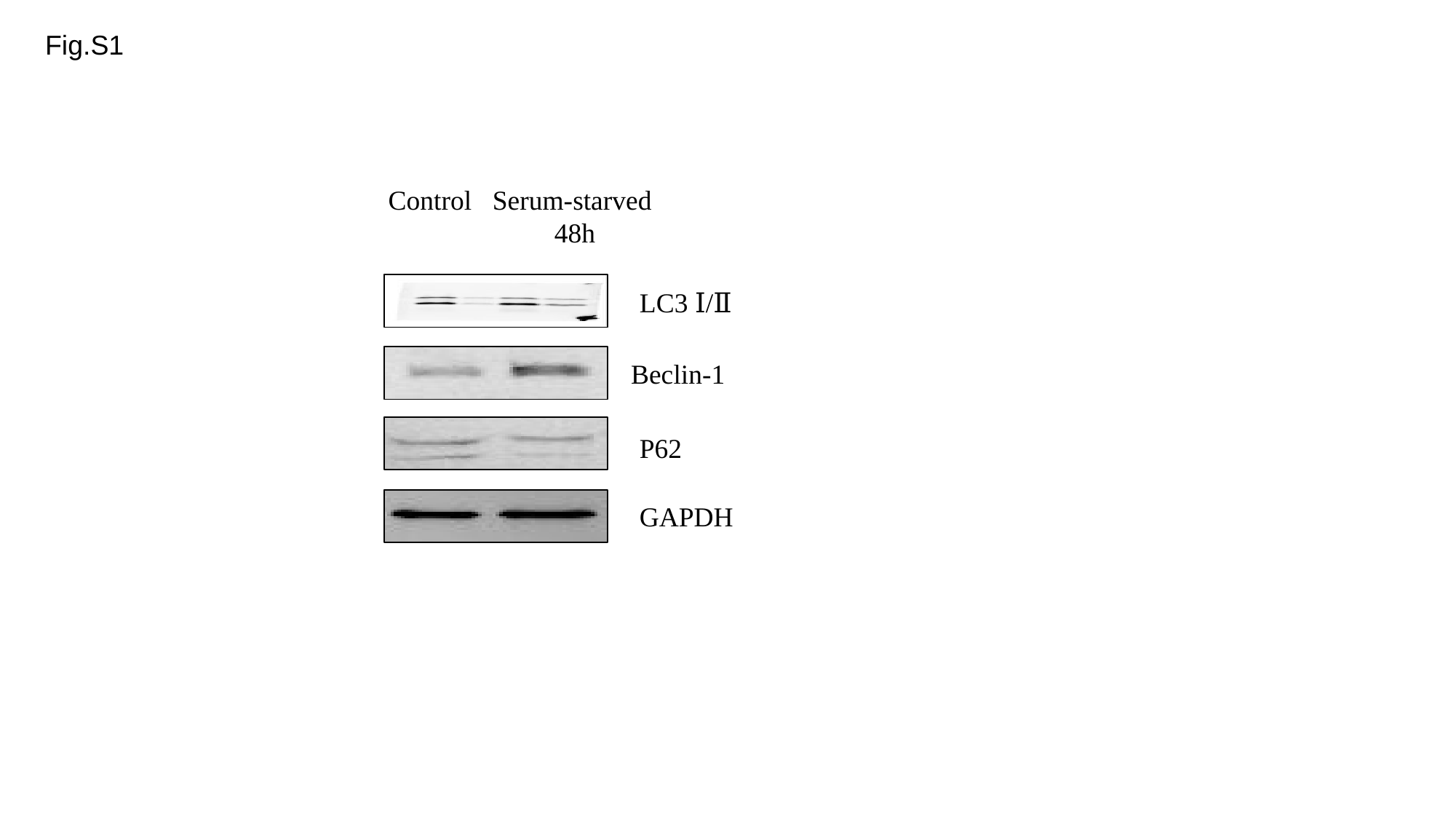

Fig.S1
Control Serum-starved
 48h
LC3 Ⅰ/Ⅱ
Beclin-1
P62
GAPDH

Supplement: Supplemental Information 7 [file peerj-08-9754-s007.pptx]

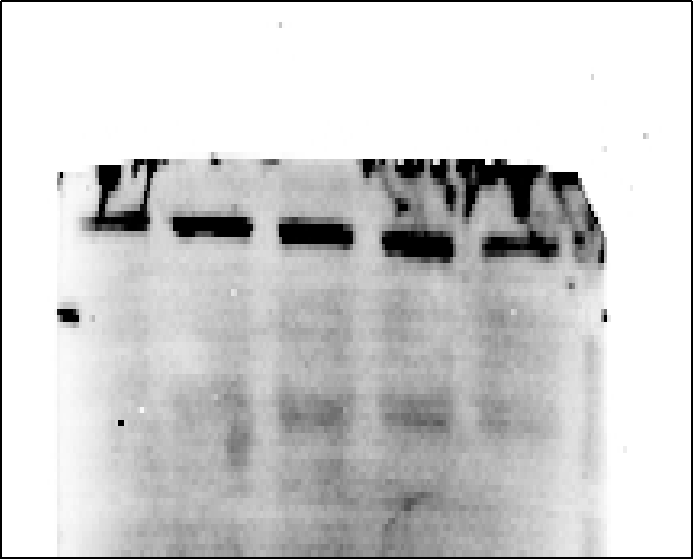

Supplement: Supplemental Information 8 [file peerj-08-9754-s008.zip › Supplemental files/Fig.4 Atg12-1.png]

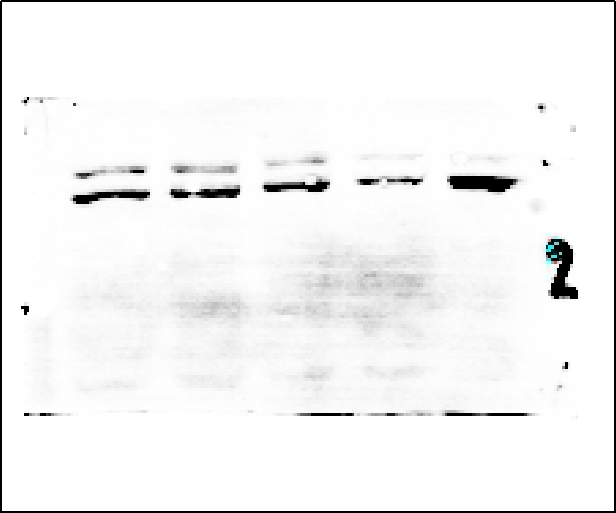

Supplement: Supplemental Information 8 [file peerj-08-9754-s008.zip › Supplemental files/Fig.4 Atg16L1-1.png]

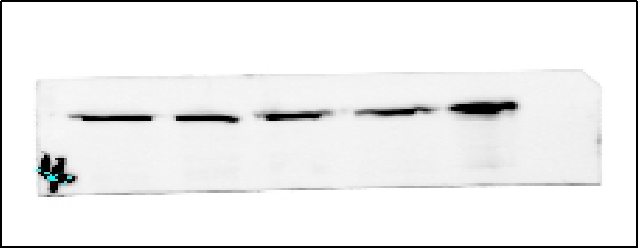

Supplement: Supplemental Information 8 [file peerj-08-9754-s008.zip › Supplemental files/Fig.4 Atg5-2.png]

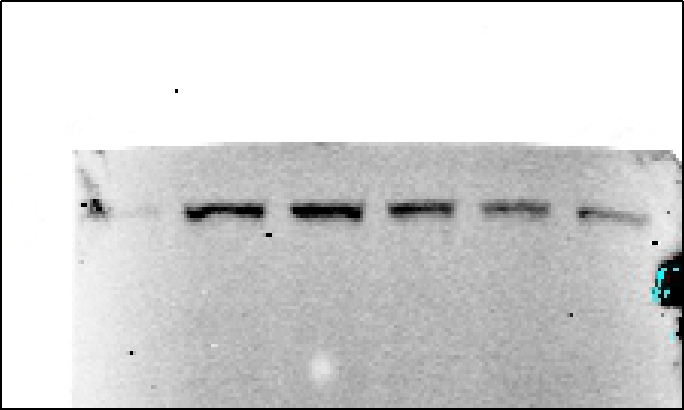

Supplement: Supplemental Information 8 [file peerj-08-9754-s008.zip › Supplemental files/Fig.4 Atg7-1.png]

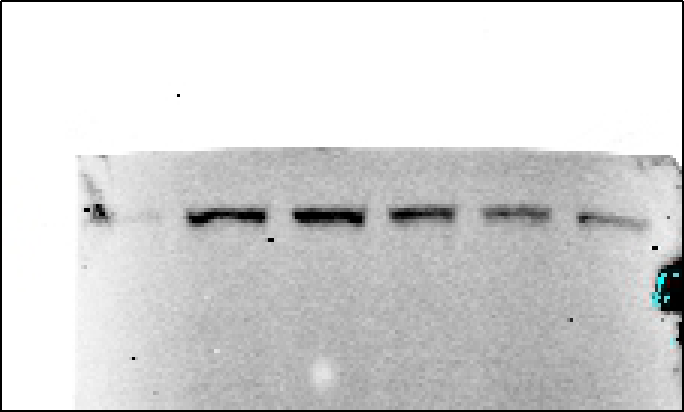

Supplement: Supplemental Information 8 [file peerj-08-9754-s008.zip › Supplemental files/Fig.4 Atg7-3.png]

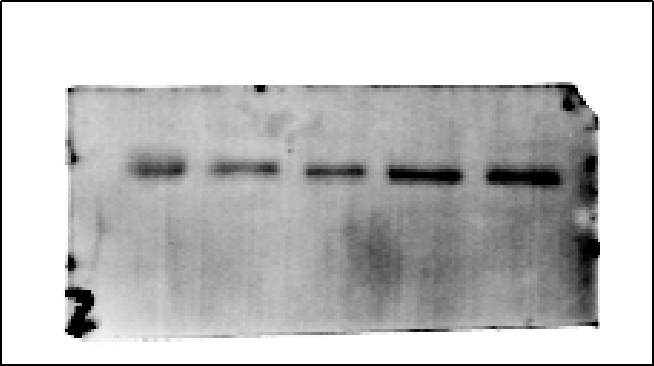

Supplement: Supplemental Information 8 [file peerj-08-9754-s008.zip › Supplemental files/Fig.4 Beclin-1-1.png]

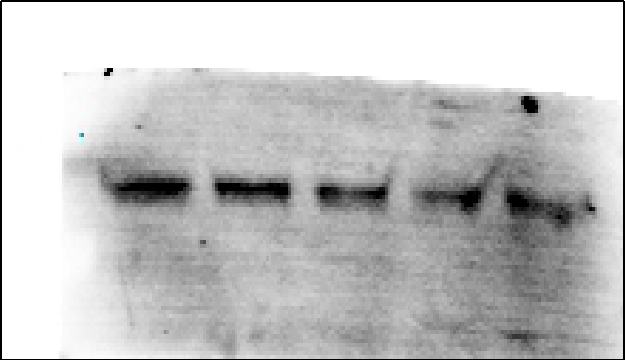

Supplement: Supplemental Information 8 [file peerj-08-9754-s008.zip › Supplemental files/Fig.4 Beclin-1-2.png]

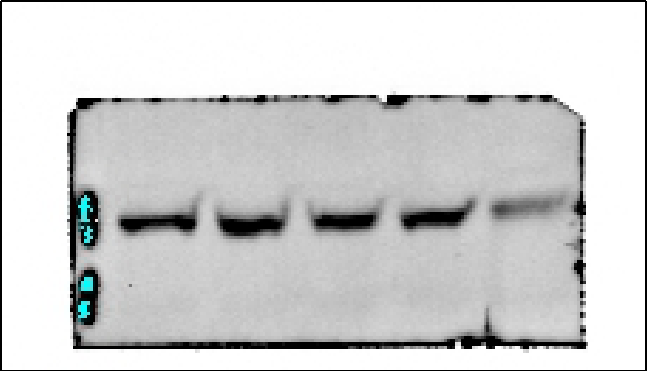

Supplement: Supplemental Information 8 [file peerj-08-9754-s008.zip › Supplemental files/Fig.4 Beclin-1-3.png]

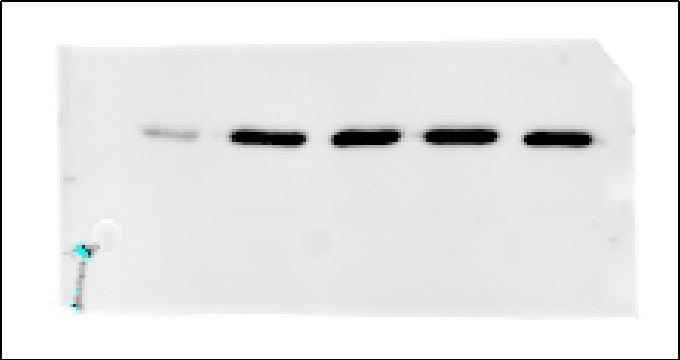

Supplement: Supplemental Information 8 [file peerj-08-9754-s008.zip › Supplemental files/Fig.4 GAPDH-1.png]

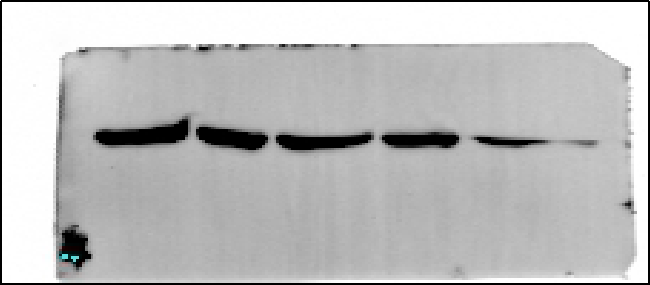

Supplement: Supplemental Information 8 [file peerj-08-9754-s008.zip › Supplemental files/Fig.4 GAPDH-2.png]

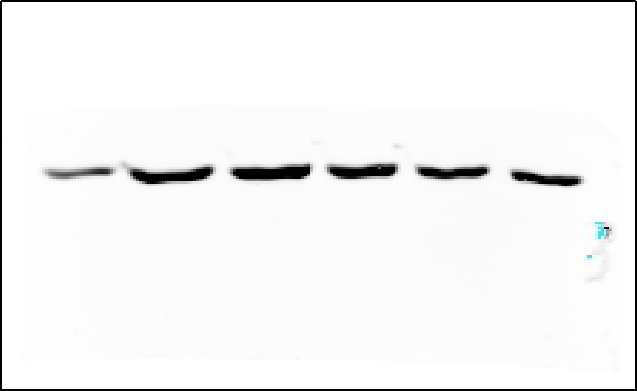

Supplement: Supplemental Information 8 [file peerj-08-9754-s008.zip › Supplemental files/Fig.4 GAPDH-3.png]

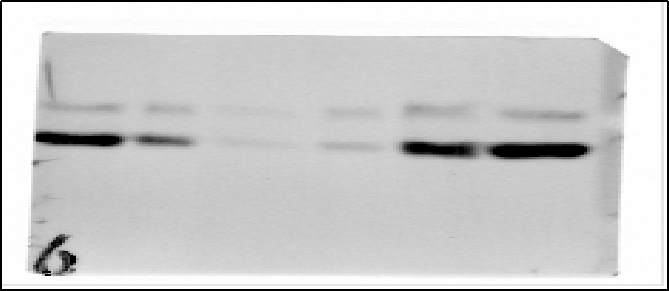

Supplement: Supplemental Information 8 [file peerj-08-9754-s008.zip › Supplemental files/Fig.4 LC3-1.png]

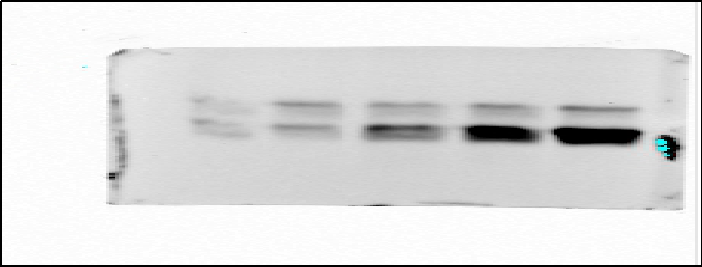

Supplement: Supplemental Information 8 [file peerj-08-9754-s008.zip › Supplemental files/Fig.4 LC3-2.png]

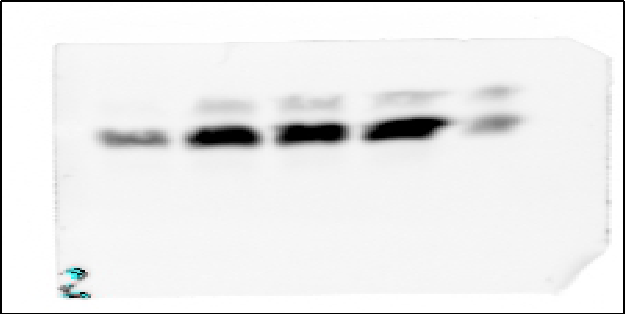

Supplement: Supplemental Information 8 [file peerj-08-9754-s008.zip › Supplemental files/Fig.4 LC3-3.png]

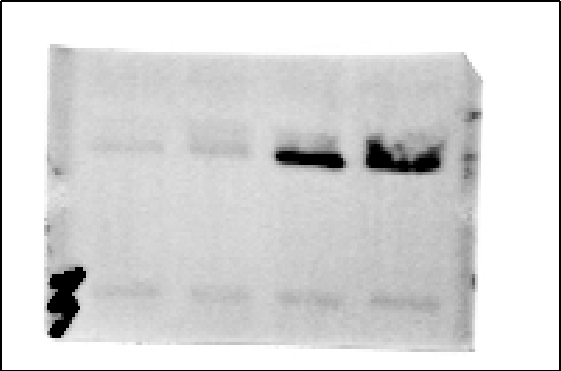

Supplement: Supplemental Information 8 [file peerj-08-9754-s008.zip › Supplemental files/Fig.4 P62-1.png]

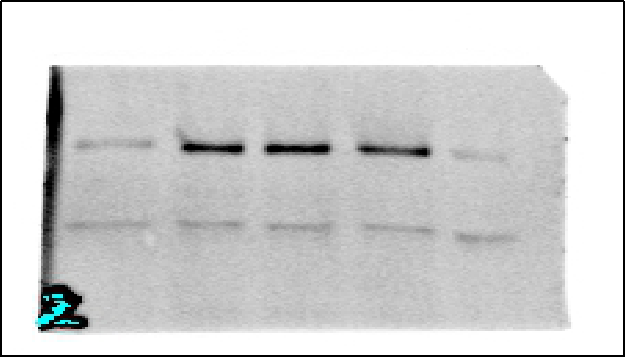

Supplement: Supplemental Information 8 [file peerj-08-9754-s008.zip › Supplemental files/Fig.4 P62-2.png]

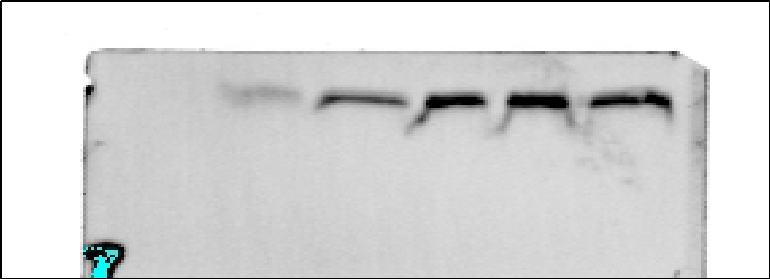

Supplement: Supplemental Information 8 [file peerj-08-9754-s008.zip › Supplemental files/Fig.4 P62-3.png]

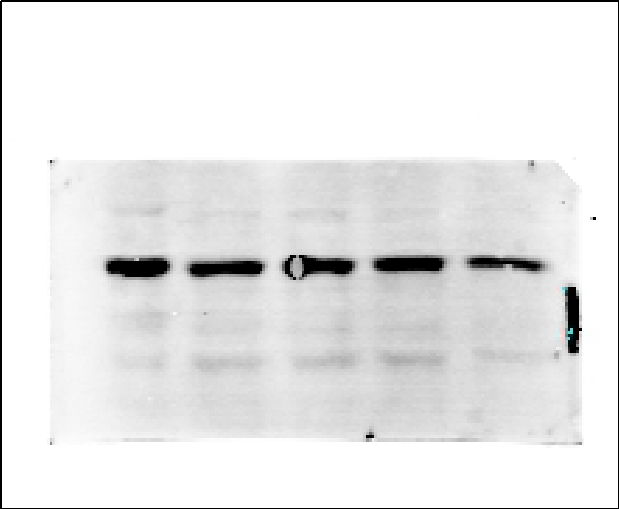

Supplement: Supplemental Information 8 [file peerj-08-9754-s008.zip › Supplemental files/Fig.4 Atg5-1.png]

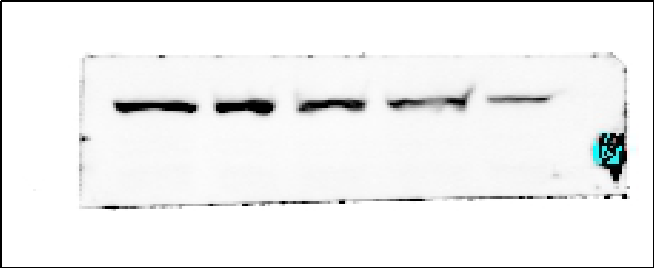

Supplement: Supplemental Information 8 [file peerj-08-9754-s008.zip › Supplemental files/Fig.4 Atg7-2.png]

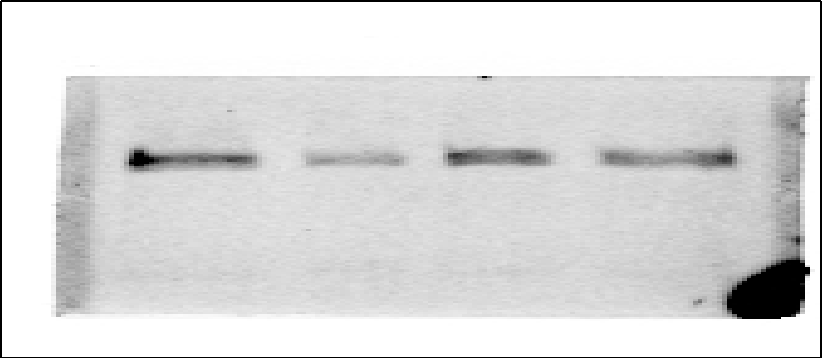

Supplement: Supplemental Information 8 [file peerj-08-9754-s008.zip › Supplemental files/Fig.S1 Beclin-1.png]

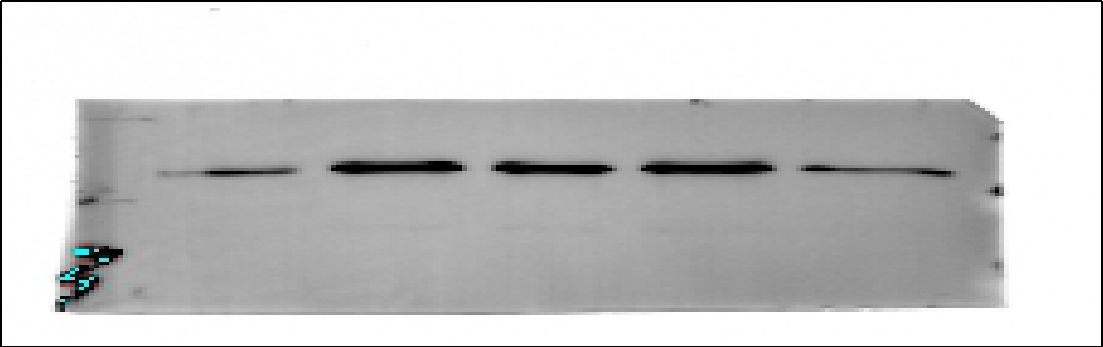

Supplement: Supplemental Information 8 [file peerj-08-9754-s008.zip › Supplemental files/Fig.S1 GAPDH-1.png]

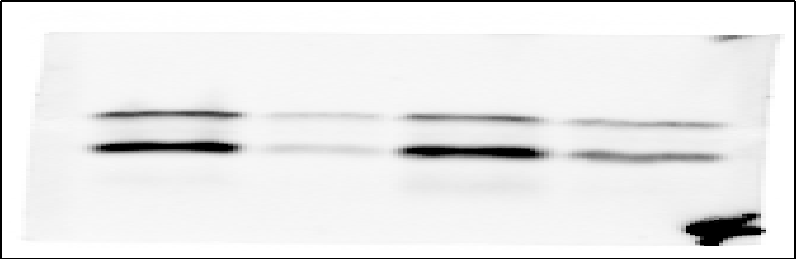

Supplement: Supplemental Information 8 [file peerj-08-9754-s008.zip › Supplemental files/Fig.S1 LC3-1.png]

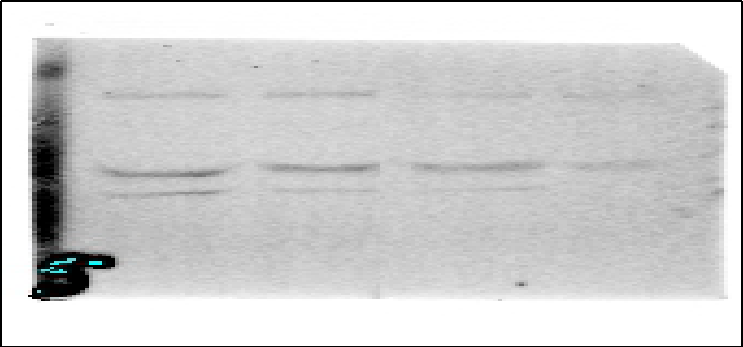

Supplement: Supplemental Information 8 [file peerj-08-9754-s008.zip › Supplemental files/Fig.S1 P62-1.png]
